# Effect of Gegen Qinlian Decoction combined with dietary management on patients with damp-heat Type-2 diabetes mellitus and metabolic syndrome

**DOI:** 10.12669/pjms.40.7.9476

**Published:** 2024-08

**Authors:** Bin Nie, Xiulan Liu, Yanni Xiao, Chang Liu, Qiong Zhao

**Affiliations:** 1 Bin Nie, Department of Childhood Psychology, The School of Clinical Medicine, Hunan University of Chinese Medicine, Brain Hospital of Hunan Province, Changsha City, Hunan Province 410007, P.R. China; 2 Xiulan Liu, Department of Emergency, The School of Clinical Medicine, Hunan University of Chinese Medicine, Brain Hospital of Hunan Province, Changsha City, Hunan Province 410007, P.R. China; 3 Yanni Xiao, Department of Scientific Research, The School of Clinical Medicine, Hunan University of Chinese Medicine, Changsha City, Hunan Province 410007, P.R. China; 4 Chang Liu, Department of Psychiatric, Brain Hospital of Hunan Province, The Second People’s Hospital of Hunan Province, Changsha City, Hunan Province 410007, P.R. China; 5 Qiong Zhao. Department of Obstetrics and Gynecology, Brain Hospital of Hunan Province, The Second People’s Hospital of Hunan Province, Changsha City, Hunan Province 410007, P.R. China

**Keywords:** Gegen Qinlian decoction, Type-2 diabetes mellitus, Dietary management, Metabolic syndrome

## Abstract

**Objective:**

To explore the effect of Gegen Qinlian Decoction (GQD) combined with dietary management in the treatment of patients with Type-2 diabetes mellitus (T2DM) and metabolic syndrome (MetS) (T2DM MetS).

**Methods:**

This is a retrospective analysis of 102 cases of T2DM in the Brain Hospital of Hunan Province, China from April 2020 to February 2023. Of them, 49 patients received conventional drug treatment (control group), and 53 patients received GQD combined with dietary management on the basis of conventional drugs (observation group). Treatment efficacy was calculated, and blood glucose levels before and after the treatment, blood lipid-related indicators, tumor necrosis factor-α (TNF-α) and adiponectin (ADP) levels, and incidence of adverse reactions were compared between the two groups.

**Results:**

The total efficacy of the observation group (92.45%) was significantly higher than that of the control group (75.51%) (*P*<0.05). After the treatment, blood glucose and lipid indicators in both groups were significantly improved compared to pretreatment levels, and were significantly better in the observation group than in the control group (*P*<0.05). After the treatment, TNF-α levels in both groups decreased compared to before the treatment, and were significantly lower in the observation group compared to the control group. Levels of ADP after the treatment increased, and were significantly higher in the observation group compared to the control group (*P*<0.05).

**Conclusions:**

When taken as an adjunct to the conventional drug regimen, GQD combined with dietary management can effectively regulate blood glucose and lipid metabolism in patients with T2DM and MetS (T2DM MetS), improve TNF-α and ADP levels, and enhance disease treatment effectiveness.

## INTRODUCTION

Metabolic syndrome (MetS) is characterized by a clustering of metabolic abnormalities, including impaired glucose tolerance, dyslipidemia, abdominal obesity and/or hypertension.^1^ It is an important risk factor for cardiovascular disease and Type-2 diabetes mellitus (T2DM).^1^,^2^ Studies show that T2DM combined with MetS is associated with high clinical risk that may seriously affect physical and mental health of patients and their overall quality of life.^2^,^3^ Currently, measures such as insulin injections, oral hypoglycemic drugs, antiplatelet aggregation drugs, and blood lipid regulators are commonly used for managing patients with T2DM and MetS (T2DM MetS).^3^,^4^ These measures can alleviate clinical symptoms to a certain extent, but the overall effect is suboptimal.^2^,^4^,^5^ In addition to pharmacological agents, dietary management is also an important way of managing T2DM with MetS.^6^

Traditional Chinese medicine (TCM) categorizes T2DM into the category of “thirst quenching”, and suggests that the onset of the disease is caused by internal and external factors that cause damage to the spleen and stomach, leading to dereliction of spleen and stomach transportation, accumulation of heat and internal storage, and consumption of fluids that damage the body.^7^,^8^ Gegen Qinlian decoction (GQD) is a commonly used formula for treating T2DM in TCM. It can detoxify and clear heat, can alleviate oxidation, has an anti-inflammatory effect, and regulates blood lipids and blood glucose.^9^

Though substantial studies have investigated the effect of Gegen Qinlian decoction for T2DM, but to the best of our knowledge, no study have investigated the combination of GQD and dietary management on patients with T2DM and metabolic syndrome. In recent years, our hospital has adopted a combination of GQD and dietary management regimen as addition to conventional pharmacological agents to treat patients with T2DM MetS. This study aimed to analyze the treatment status of patients, and to clarify the therapeutic value of GQD combined with dietary management plan.

## METHODS

A total of 102 patients with T2DM MetS (53 males and 49 females), admitted to the Brain Hospital of Hunan Province from April 2020 to February 2023, were retrospectively selected. A total of 49 patients received conventional Western medicine treatment and were set as the control group, while 53 patients received a combination of GQD and dietary management on the basis of conventional drugs, and were designated as the observation group.

### Ethical approval

The ethics committee of Brain Hospital of Hunan Province approved this study on July 31, 2023, No. 2021-031.

### Inclusion criteria:

- Met the diagnostic criteria for T2DM.^10^
- Met MetS diagnostic criteria.^11^
- Classified as “dampness heat” syndrome based on the TCM classification.^12^
- Patients aged > 40.
- The clinical data is complete.

### Exclusion criteria:

- Patients with ketoacidosis and other diabetes related complications
- Lactating or pregnant women.
- Patients with Type-1 diabetes mellitus.
- Patients with malignant tumors.
- Patients with organic lesions such as gastrointestinal and renal liver.
- Patients with autoimmune, hematopoietic system and infectious diseases.
- Patients with mental system disorders.

### Treatment methods

Patients in the control group received conventional medication treatment, while the patients in the observation group received GQD combined with dietary management on the basis of conventional drug treatment.

### Conventional medication treatment

After admission, all patients were given health education (including basic understanding of the diseases, smoking cessation and alcohol restriction, exercise guidance, dietary guidance, etc.), as well as lipid-lowering and antihypertensive measures. Symptomatic treatment included atorvastatin (Pfizer Pharmaceuticals, 20 mg/tablet, one tablet per day) and metformin (Beijing Jingfeng Pharmaceutical Group Co., Ltd.; 0.5g/tablet, three doses per day, 0.5 g/dose).

### GQD

*Dosage of each ingredients:* Scutellaria baicalensis 22.5 g, Coptis chinensis 22.5 g, Pueraria lobata 60 g, 3.5 g of dried ginger, and one gram of raw licorice. The decoction was diluted in water in the Chinese Medicine Department of our hospital, and divided into portions, with a total of 200mL per portion. Patients took the decoction twice a day, once in the morning and once in the evening, 200mL each time.

### Dietary management

Dedicated nursing staff conducted dietary health assessments on patients and established personal records; Food exchange method dietary software (Shenzhen Jianyue Life Technology Co., Ltd., China) was used to analyze patient’s personal information, and personalized calorie charts were developed based on the patient’s height, body mass, work nature, etc. Patients selected menu options provided by the software to achieve a reasonable combination of three meals a day. Nursing staff who have received professional training participated in educating and transferring disease-related knowledge to patients or guardians. Simple cooking tips guidance was provided for the selected recipes. Daily diet choices made by patients were recorded and printed out. Patient were instructed to continue eating according to the software-provided guidelines after the discharge.) After the discharge, patients were followed-up by telephone and WeChat.

Patients in both groups were treated for three months.

### Observation indicators:

### Effect

***Significant effect:*** the disappearance of symptoms such as thirst, polydipsia, and polyuria, as well as the restoration of normal blood glucose levels and pancreatic β cell function; *Effective:* The improvement of symptoms such as thirst, polydipsia, and polyuria, as well as the recovery of blood glucose levels and pancreatic β cell function but not reaching normal levels; *Invalid:* Failure to meet the above standards was considered. Significant effects and efficacy were included in the total effective rate.

Blood glucose levels, including abdominal blood glucose (FPG), 2-hour postprandial blood glucose (2hPG), glycosylated hemoglobin (HbA1c), and insulin resistance index (HOMA-lr), were measured. Hitachi 7600 fully automatic biochemical analyzer (Hitachi, Japan) and supporting reagent kits were used to detect FPG, 2hPBG, and HbA1c levels. Detection of insulin (FIN) was done using electrochemical immunoluminescence (Roche, Switzerland), HOMA-IR=FPG × FIN/22.5.

Levels of blood lipid indicators, such as low-density lipoprotein cholesterol (LDL-C), high-density lipoprotein cholesterol (HDL-C), total cholesterol (TC), and triglycerides (TG), were measured. Enzymatic determinationwas done by the reagent kit, purchased from Shanghai Enzyme-linked Biotechnology Co., Ltd.

Serum TNF-α and ADP levels were determined by enzyme-linked immunosorbent assay using the reagent kit from Shanghai Enzyme-linked Biotechnology Co., Ltd.

The incidence of adverse reactions was also noted.

### Statistical analysis

All data analysis was conducted using SPSS 25.0 software (IBM Corp, Armonk, NY, USA). The normality of the data was evaluated using the Shapiro-Wilk test. The data of normal distribution was represented by mean ± standard deviation, independent sample *t*-test was used for inter group comparison, and paired *t*-test was used for intra group comparison before and after. Non-normal distribution data were represented by median and interquartile intervals. Mann Whitney *U* test was used for inter group comparison, and the Wilcoxon signed rank test was used for intra group comparison before and after the treatment. The counting data were represented by the number of cases using chi square test. *P*<0.05 indicated statistically significant difference.

## RESULTS

This study included 102 patients, 49 in the control group and 53 in the observation group. Age range of the patients was 42 to 77 years, with a mean age of 61.04 ± 8.12 years; the BMI was 25.3~35.6kg/m2, with a mean of 29.59 ± 2.47kg/m2; the course of the disease was 2-9 years, with a median course of five years. There was no statistically significant difference in baseline data between the two groups of patients (*P*>0.05) (Table-I). The total efficacy of the observation group (92.45%) was significantly higher than that of the control group (75.51%) (*P*<0.05) (Table-II).

**Table-I:** Comparison of baseline data between two groups.

Group	n	Gender (male/female)	Age (years)	BMI (kg/m^2^)	Course of disease (years)
Observation group	53	30/23	60.81±8.38	29.24±2.28	5.5(5, 6)
Control group	49	23/26	61.29±7.91	29.97±2.64	5(5, 6)
*χ^2^/t/Z*		0.953	-0.294	-1.495	-1.003
*P*		0.329	0.770	0.138	0.316

**Table-II:** Comparison of treatment effects between two groups.

Group	n	Significant effect	Effective	Invalid	Total effective rate
Observation group	53	27 (50.94)	22 (41.51)	4 (7.55)	49 (92.45)
Control group	49	17 (34.69)	20 (40.82)	12 (24.49)	37 (75.51)
*χ^2^*					6.221
*P*					0.045

Before the treatment, there was no statistically significant difference in blood glucose levels between the two groups (*P*>0.05). After the treatment, levels of FPG, 2 hPG, HbA1c, and HOMA-lr of both groups significantly decreased, and were significantly lower in the observation group compared to the control group (*P*<0.05) (Table-III).

**Table-III:** Comparison of blood glucose levels between two groups.

Time	Group	n	FPG (mmol/L)	2 hPG (mmol/L)	HbA1c (%)	HOMA-lR
Before treatment	Observation group	53	8.06±0.71	12.28±1.70	8.29±0.83	4.51±0.81
	Control group	49	7.97±0.88	12.52±1.75	8.36±0.88	4.57±0.63
	*t*		0.588	-0.706	-0.453	-0.431
	*P*		0.558	0.482	0.652	0.668
After treatment	Observation group	53	5.95±0.64*	7.83±1.52*	6.72±0.84*	2.67±0.52*
	Control group	49	6.55±1.41*	8.47±1.64*	7.61±0.81*	3.55±0.53*
	*t*		-2.770	-2.046	-5.469	-8.404
	*P*		0.007	0.043	<0.001	<0.001

* , P<0.001 when compared with the same group before treatment.

Before the treatment, there was no statistically significant difference in the levels of blood lipid indicators between the two groups (*P*>0.05). Treatment led to a significant decrease in the levels of LDL-C, TC, and TG in both groups, and these posttreatment levels were significantly lower in the observation group compared to the control group(*P*<0.05). HDL-C level increased in both groups compared to pretreatment levels, and were significantly higher in the observation group than in the control group (*P*<0.05) (Table-IV).

**Table-IV:** Comparison of blood lipid index levels between two groups (mmpl/L).

Time	Group	n	LDL-C	HDL-C	TC	TG
Before treatment	Observation group	53	3.35±0.42	0.78(0.63, 0.84)	4.80±0.62	2.31±0.27
	Control group	49	3.46±0.49	0.82(0.65, 0.87)	4.60±0.69	2.29±0.29
	*t/Z*		-1.239	-1.173	1.529	0.450
	*P*		0.218	0.241	0.129	0.654
After treatment	Observation group	53	2.27±0.25*	1.41±0.25*	3.65±0.23*	1.45±0.19*
	Control group	49	2.58±0.30*	1.21±0.26*	4.01±0.30*	1.78±0.21*
	*t*		-5.607	3.975	-6.807	-8.306
	*P*		<0.001	<0.001	<0.001	<0.001

* , P<0.001 when compared with the same group before treatment.

Levels of TNF-α and ADP were in the two groups before the treatment (*P*>0.05). After the treatment, there was a significant decrease in the levels of TNF-α in both groups. TNF-α levels were markedly lower in the observation group compared to the control group(*P*>0.05). The post treatment ADP levels increased significantly, and were markedly higher in the observation group compared to the control group (*P*<0.05) (Table-V). There was no statistically significant difference in the incidence of adverse reactions between the observation group (7.54%) and the control group (4.08%) (*P*>0.05) (Table-VI).

**Table-V:** Comparison of TNF-α and ADP levels between two groups.

Time	Group	n	TNF-α (ng/L)	ADP (mg/L)
Before treatment	Observation group	53	178.64±19.68	5.72±0.70
	Control group	49	181.71±22.56	5.82±0.64
	*t*		-0.734	-0.721
	*P*		0.464	0.473
After treatment	Observation group	53	85.96±12.12*	7.03±0.70*
	Control group	49	103.35±12.79*	6.23±0.76*
	*t*		-7.048	5.581
	*P*		<0.001	<0.001

* , P<0.001 when compared with the same group before treatment.

**Table-VI:** Comparison of adverse reaction rates between two groups.

Group	n	Diarrhea	Vomit	Nausea	Total occurrence rate
Observation group	53	2 (3.77)	0 (0.00)	2 (3.77)	4 (7.54)
Control group	49	0 (0.00)	1 (2.04)	1 (2.04)	2 (4.08)
*χ^2^*					0.552
*P*					0.457

## DISCUSSION

The results of this study indicate that the combination of GQD and dietary management for patients with T2DM MetS is safe and can more effectively regulate blood lipid and blood glucose levels, and improve disease treatment effectiveness compared to the conventional Western medicine approach alone. Liu H et al.^13^ confirmed that providing dietary guidance and GQD during routine treatment for patients with T2DM is beneficial for regulating blood glucose levels, which is consistent with the results of our study.

He L et al.^14^ also confirmed that GQD has an important auxiliary role in the treatment of T2DM, and is beneficial for regulating glucose and lipid metabolism, improving disease prognosis. Moreover, He et al^14^ pointed out that Scutellaria baicalensis, Pueraria lobata, and Coptis chinensis all have significant anti-inflammatory and antipyretic effects, and can effectively inhibit thrombosis, regulate immune function, and reduce the degree of oxidative stress in the body. Ren L et al.^15^ also confirmed that GQD can enhance the hypoglycemic effect of metformin without causing serious adverse reactions. Huang Z et al.^16^ implemented auxiliary intervention with GQD on MetS patients, and showed that the levels of blood glucose indicators (FPG, 2 hPG, HbA1c) and blood lipid indicators (LDL-C, HDL-C, TC, TG) in the patients were better than those in the control group. Their results also showed benefits of combined regimen for improving the obesity level of MetS patients. A healthy diet is also an important influencing factor in ensuring the outcome of T2DM combined with MetS disease.^17^,^18^

Moutou KE et al.^17^ showed that long-term standardized, scientific, and healthy diet not only allows patients to effectively control their weight, regulate lipid metabolism disorders and high blood glucose, but also reduces the requirement and dosage of medication. Alzahrani AH et al.^18^ also confirmed that intervention with a self-made low carbohydrate and high protein diet plan in patients with T2DM is beneficial for regulating their blood lipid levels, downregulating the content of inflammatory factors, and reducing the risk of cardiovascular disease.

As shown by Chen Z et al.,^19^ ADP, an insulin sensitizing hormone produced by adipocytes, plays an important role in the pathogenesis and progression of insulin resistance and diabetes. It suppresses inflammation and atherosclerosis, promotes the uptake and metabolism of free fatty acids in circulating blood, liver and muscle tissue, reduces the levels of TG and free fatty acids and alleviates insulin resistance. Additionally, ADP can prevent the proliferation of monocyte precursor cells and have a negative regulatory effect on the expression of TNF-α, thereby reducing the degree of inflammatory response.^19^

Imanparst F et al.^20^ have shown that TNF-α can inhibit insulin receptor self-phosphorylation, block insulin-stimulated glucose uptake, and downregulate the number of insulin receptors, thereby inducing hyperinsulinemia and exacerbating insulin resistance. TNF-α can also regulate the activity of lipoprotein lipase, stimulate the generation of free fatty acids, and inhibit fatty acid absorption, increase TG, LDL-C, TC synthesis, and reduce HDL-C synthesis.^21^ This study investigated and analyzed the levels of TNF-α and ADP in patients with T2DM MetS. Our results showed that the combination of GQD and dietary management led to significantly better serum TNF-α and ADP levels compared to conventional treatment regimen.

### Limitations

This is a retrospective study with a small sample size, which may limit the extrapolation of results. Additionally, the duration of treatment for patients in our study was relatively short. Further long-term efficacy study is needed to confirm the efficacy of the combination of GQD and dietary management in the treatment of patients with T2DM MetS. Finally, although TCM syndromes are evaluated by professionally trained doctors, there is still subjectivity that may lead to biased results.

## CONCLUSION

Combination of GQD and dietary management in addition to conventional pharmacological agents is safe and can effectively regulate blood glucose and lipid metabolism in patients with T2DM MetS, improve TNF-α and ADP levels, enhance disease treatment effectiveness.

### Authors’ contributions:

**BN and XL:** Conceived and designed the study.

**YX, CL and QZ:** Collected the data and performed the analysis.

**BN and XL:** Were involved in the writing of the manuscript and is responsible for the integrity of the study.

All authors have read and approved the final manuscript.
